# Investigation of Igf-1, Igf-Bp3 and Igf-Bp5 levels in umbilical cord blood of infants with developmental dysplasia of the hip

**DOI:** 10.55730/1300-0144.5628

**Published:** 2023-02-28

**Authors:** Esra DEMİREL, Eyüp ŞENOCAK, Gamze Nur CİMİLLİ ŞENOCAK, Ali ŞAHİN, Berrin GÖKTUĞ KADIOĞLU, Özlem GÜNDÜZ

**Affiliations:** 1 Department of Orthopedics and Traumatology, Erzurum Training and Research Hospital, Erzurum, Turkey; 2 Department of Obstetrics and Gynecology, Faculty of Medicine, Atatürk University, Erzurum, Turkey; 3 Department of Orthopedics and Traumatology, Ankara City Hospital, Ankara, Turkey; 4 Department of Obstetrics and Gynecology, Erzurum Training and Research Hospital, Erzurum, Turkey

**Keywords:** IGF-1, IGF-BP3, IGF-BP5, developmental hip dysplasia, umbilical cord blood

## Abstract

**Background/aim:**

IGF-1 (insulin-like growth factor-1) is an important regulator of bone formation. Its deficiency has been associated with fetal growth disorders and hip dysplasia. The aim of this study was to evaluate whether IGF-1, IGF-BP3 (insulin like growth factor-binding protein 3), and IGF-BP5 levels in the umbilical cord blood can be predictive for early diagnosis of DDH.

**Materials and methods:**

Umbilical cord blood samples were collected from 860 mothers with pregnancies at high risk for DDH between October 2020 and January 2021. Mothers at 37–42 weeks of gestation, with risk factors for DDH, who delivered healthy infants were included. Blood samples were collected during delivery. Each eligible infant was medically followed up and underwent a hip ultrasound in the postnatal 2nd or 3rd month. Infants diagnosed with DDH were matched with a healthy cohort in terms of sex, birth weight, maternal age, and gestational week, and the IGF-1, IGF-BP3 and IGF-BP5 levels were studied and compared.

**Results:**

Evaluation was made of 20 infants diagnosed with DDH and 60 healthy infants. Of the total 80 infants, 72.5% were female. The umbilical cord blood levels of IGF-1 and IGF-BP3 were similar in both groups. The IGF-BP5 values were significantly lower in the DDH patient group. Except for DDH diagnosis, the other categorical variables of the study did not appear to influence the levels of any of the IGFs.

**Conclusion:**

Umbilical blood samples could potentially help diagnose DDH. The levels of IGF-BP5 were shown to be significantly lower in infants with DDH.

## 1. Introduction

Developmental dysplasia of the hip (DDH), which affects 1–5 of every 1000 infants, is characterized by structural abnormalities in which the relationship of the femoral head and the acetabulum is impaired during the development of the joint [1–3]. Hip dysplasia is the most common cause of hip osteoarthritis in women aged <40 years, accounting for 5%–10% of all hip replacements in the United States [4]. Female sex, a positive family history of DDH, breech position, primiparity, swaddling, and oligohydramnios are known risk factors [1, 5–7]. The development of DDH is also thought to be affected by genetic, mechanical, and environmental factors. In recent years, genetic studies focusing on connective tissue malformation, osteogenesis, chondrogenesis, cell growth, and differentiation have been emphasized [1].

Fetal and postnatal growth is controlled by endocrinological and genetic components that combine and regulate cellular proliferation, differentiation, and apoptotic processes in target tissues, especially in the epiphysial plates [8]. The necessary substrates for this during the fetal period are provided by the mother and transferred by the placenta. This maternofetal metabolism is also regulated by endocrine factors and insulin-like growth factors (IGFs), together with other hormones [9]. IGF-1, the main insulin-like growth factor, which is critical for development during the third trimester and in the first six months after birth, is also an important regulator of bone and cartilage formation. Its deficiency has been associated with fetal growth disorders [10, 11], among which is hip dysplasia [11, 12].

In biological fluids, IGFs are bound and transported by members of the IGF-BP (IGF binding protein) family and they are also thought to mediate IGF-independent actions through their own receptors [13]. IGF-1 produced in the liver is transported to target tissues such as muscle, bone and adipose tissue by binding to IGF-BP3. The main IGF-BP in human serum is growth hormone-linked IGF-BP3, which is also produced in vitro by human osteoblasts [14]. IGF-BP5 is the most abundant binding protein stored in the bone, and acts as a growth factor, increasing bone formation [15].

DDH is easier to treat when diagnosed early and is more likely to cause disability if detected at a later stage. The goal of screening is to prevent late diagnosis of DDH, especially after 6 months postpartum. Physical examination, radiography, and ultrasonography all play a role in scanning for DDH [7, 16, 17], but despite these efforts to recognize the condition as early as possible after birth, the diagnosis is often delayed, especially in populations with a high fertility rate but limited access to medical care [18–20]. The objective of this study was to evaluate whether IGF-1, IGF-BP3 and IGF-BP5 levels in the umbilical cord blood can be predictive for early and accurate diagnosis of DDH. Given their importance in connective and bone tissue growth, it was hypothesized that different levels of IGFs would be correlated with DDH and could therefore be used as screening tests during the first days postpartum.

## 2. Materials and methods

### 2.1. Patient selection

This prospective observational study was approved by the Local Institutional Review Board. The study included infants born between October 2020 and January 2021, at our tertiary center of Erzurum Training and Research Hospital, with the informed and written consent of their parents. The study population consisted of consecutive mothers who gave birth at our institution between the aforementioned dates (through a cesarean section (C/S) or normal vaginal delivery (NVD)), were at 37–42 weeks of gestation, showed risk factors for DDH, and gave birth to healthy infants. Female infants, first births, breech births, history of DDH in first-degree relatives, and the presence of oligohydramnios were regarded as risk factors for DDH. The study exclusion criteria were defined as stillbirth, infant mortality within six months of the birth, congenital anomalies, twins, the presence of gestational diabetes, preeclampsia or placental pathology, or small (SGA) or large (LGA) according to gestational age newborns.

### 2.2. Blood sample collection

After double clamping the umbilical cord at the placental end during delivery (C/S or NVD), 5 cc of blood was taken from the umbilical vein into anticoagulant-free tubes and kept at room temperature for about 30 min. Next, in accordance with the kit instructions (USCN, China, Cat. no: SEC659Hu), the sample was centrifuged at 1000 × *g* for 20 min at +4° C. The obtained serum was transferred into Eppendorf tubes and kept at −80 °C until it could be processed. The kit was available at our Institution for a limited period of time. A total of 860 samples were collected during the study dates. IGF-1, IGF-BP3, and IGF-BP5 levels were studied from the blood samples of the infants included in the study.

### 2.3. Radiological and medical follow-up

All infants born between the aforementioned dates were prospectively followed up and their cord blood was stored as new patients became eligible. A routine physical examination and hip ultrasound (7.5 mm/Hz linear probe, Fukuda Denshi Co. Ltd, Tokyo, Japan) were performed between the second or third month after birth. Angle measurements were performed electronically on digital images and classified according to the Graf classification. All subtypes of the Graf classification were included in the study and treatment was planned accordingly. Infants diagnosed with DDH constituted the patient group. Their blood samples, collected at birth, were sorted and separated from the rest. The process continued as the number of diagnosed patients increased. During follow-ups, 39 infants who were diagnosed with genetic, cardiac, endocrinological or renal pathologies and 1 infant who died due to sepsis after birth were excluded from the study. At the end of the study period, 20 infants had been diagnosed with DDH, and they constituted the study group. They were all initially treated with a Tubingen abduction orthosis. All but one required no further treatment, while one patient underwent a closed reduction procedure in the operating room.

A healthy cohort, at a ratio of 1:3 was used as a control group (n = 60). This cohort consisted of infants who matched the study group in terms of sex, maternal age, and gestational week, and was randomly selected, using a randomization table, from the remaining patient samples. Finally, once the study groups had been defined, their umbilical cord blood levels of IGF-1, IGF-BP3, and IGF-BP5 were analyzed and compared between the groups.

### 2.4. Statistical analysis

All data were analyzed using SPSS software for Windows (v.25.0, IBM Corp, Chicago, IL, USA). Normality tests were used to evaluate normal distribution and kurtosis skewness values. During data analysis, the independent sample t-test was used to compare quantitative data with normal distribution. In the absence of normal distribution, the Mann–Whitney U test was used for two groups and the Kruskal–Wallis H test for more than two groups. Categorical data comparison was performed using the Pearson chi-squared test. Post hoc power analysis was performed with G. Power-3.1.9.2 software to assess the strength of the obtained results. A p-value of <0.05 was considered statistically significant.

## 3. Results

A total of 1386 live deliveries occurred in the defined study period. After applying the inclusion and exclusion criteria, 860 mothers with a high risk of DDH were identified. Of these infants, 39 were diagnosed with genetic, cardiac, endocrinological or renal pathologies, 1 died due to sepsis and 26 were lost to follow-up. Of the remaining 794 patients, 20 were diagnosed with DDH (Figure). They were matched at a ratio of 3:1 with a healthy cohort in terms of sex, maternal age, and gestational week. Of the 774 healthy infants, 60 were selected for the control group.

A total of 80 infants, 72.5% female and 23.3% male, were included in the final analysis. Mean birth weight, gestational age, maternal age, infant sex and data regarding primigravida/multiparity were similar in both groups. Of the 20 patients diagnosed with DDH, 13 (65%) were type 2b, 6 (30%) were type 2c, and one was type 3 (type 1: centered normal hip, type 2a: centered but physiologically immature; type 2b: centered but with maturational deficit; type 2c: centered but critical hip; type 3: dislocated hip; type 4: dislocated high hip). All baseline demographic data and their comparisons are presented in Table 1.

The IGF-1, IGF-BP3, and IGF-BP5 values of the healthy and DDH infants were compared. No differences were determined between the groups in respect of IGF-1 and IGF-BP3. The IGF-BP5 values were significantly lower in the DDH group. The relevant data are presented in Table 2.

In infants with hip dysplasia, no significant differences were detected in terms of IGF-1, IGF-BP3, and IGF-BP5 levels and the other categorical variables (sex, primigravida/multiparity data, DDH subtype and side). The data are presented in Table 3.

Since this was the first study comparing IGF values in DDH patients, a post hoc power analysis was performed to assess the strength of the obtained results. The IGF-1, IGF-BP3, and IGF-BP5 (ng/mL) values were compared between the control group infants and those diagnosed with DDH, and the strength of the results of each variable, with an alpha value of 0.05, was found to be 0.99%, 0.97%, and 0.72%, respectively. A post hoc power analysis showed that the results of the study had power of 0.72%–0.99%. The strength of the results was determined to be optimal to be able to safely comment on the outcome and the sample size was deemed appropriate.

## 4. Discussion

The results of this study showed that analyses obtained from umbilical blood samples could potentially help diagnose future conditions. Low levels of IGF-BP5 were shown to be significantly lower in infants diagnosed with hip dysplasia. This could potentially have a real clinical impact in early screening, especially in regions where access to medical care is limited and follow-ups are not always possible. Not all infants in developing countries currently have access to hip ultrasonography and screening through a blood sample could provide a game-changing opportunity for the diagnosis of debilitating conditions such as DDH. While the molecular pathways leading to the current findings are still unknown, it can be considered that this might be a starting point for better-equipped and better-financed centers to further the research on this topic, since, to the best of our knowledge, this is the first study reporting a relationship between IGFs and DDH.

Clinical and experimental studies have demonstrated that insulin-like growth factors play an important role in both fetal and postnatal growth and development [21, 22]. Linear growth, lean bone mass, and height of infants have been shown to be associated with serum IGF-1 and IGF-BP3 levels [21, 22]. IGF-1 and IGF-BP5 are also necessary for cartilage development and their balance may affect cartilage development [23–25]. In a study examining the fetal and postnatal period of mice, IGF-BP5 and IGF-1 expressions were detected in the germinal, proliferative, and hypertrophic cell layers of the acetabulum and femoral head epiphysis cartilage [15]. IGF-BP5 has also been shown to be a potential factor associated with bone metabolism, increasing the osteogenic differentiation potential of mesenchymal stem cells [26]. While the levels of IGF-BP5 were lower in the umbilical cord blood of the current study patients with DDH, the exact mechanism of this finding is still not clear. IGF pathways stimulate the synthesis of type I collagen, which is thought to play a role in the etiopathogenesis of DDH [27, 28]. Further studies will be required to enlighten this topic.

In addition to local dysplasia and acetabular abnormalities around the hip joint, morphological pathologies of the entire pelvis are frequently present in patients with DDH [29]. In an experimental study by Chen et al., it was reported that proteins associated with IGF were positively expressed in rat hips during the development phase, which may indicate that they play a role in hip development [15]. In the light of this information, the aim of the current study was to investigate the relationship between IGF-1, IGF-BP3, and IGF-BP5 levels in umbilical cord blood of DDH infants, and the results showed that while levels of IGF-1 and IGF-BP3 were similar to those of the control group, there was a significant decrease in IGF-BP5 levels in DDH patients.

In the IGF-BP family, IGF-BP5 is the most abundant IGFBP stored in the bone and can serve as a growth factor to increase bone formation [30]. IGF-BP5 was measured in umbilical cord blood for the first time in a previous study and a positive correlation was found between IGF-BP5 and birth weight and length [31]. Kiepe et al. reported that IGF-BP5 increased growth plate chondrocyte differentiation through an IGF-I-linked mechanism, and that IGF-BP5 had a role in upregulating the IGF effect during in vivo chondrocyte differentiation [24]. In the current study, the cord blood IGF-BP5 level was significantly lower in infants with DDH compared to normal infants but no relationship was determined between these levels and other categorical study variables (such as sex, primigravida/multiparity data, DDH subtype, and laterality). The findings of this study support previous animal experiment studies which have investigated the relationship between IGF-BP5 and DDH. In two different animal studies, Chen et al. stated that decreased PAPP-A2 gene expression may be associated with DDH since it plays an important role in up- and downgrading IGF expression [15, 32]. The current study results showed a decrease in IGF-BP5 levels associated with a DDH diagnosis.

Despite the promising results, this study had some limitations. First of all, due to the limited infrastructure to store cord blood, it was decided to include only pregnancies at high risk of DDH. This might have led to a selection bias, but it is also well known that DDH is rare in the absence of risk factors [33]. Second, blood analyses of the parents were not performed. The differences mentioned in this study could have been physiological, and only related to individual characteristics or influenced by other environmental factors. Given the relatively low number of the patient cohort, it is difficult to comment on this topic. A further limitation was that the markers mentioned in this study are implicated in somatic growth at different levels and they could have been expressed more (or less) according to the growth status of the patient. Moreover, it is not known if these markers remained at the same different levels while the newborns grew, as the blood tests were not repeated during follow-up. This would have led to additional ethical problems but also social ones with the newborns’ parents. Additionally, the aim of this study was to research the validity of the studied biomarkers as a test withdrawn from the umbilical cord blood. Describing the course of Igf-1, Igf-Bp3, and Igf-Bp5 in newborns was outside the study purpose, but it is hoped that these results will arouse curiosity on this topic for future studies. Despite these limitations, this study can be considered of value as the first to report a relationship between low levels of IGF-BP5 and DDH diagnosis. A post hoc power analysis showed that the results of the study had power of 0.72%–0.99%.

In conclusion, umbilical blood samples could potentially help diagnose DDH. Low levels of IGF-BP5 were shown to be significantly lower in infants with DDH. This could potentially have a real clinical impact in early screening, especially in regions where access to medical care is limited and follow-ups are not always possible.

## Figures and Tables

**Figure:**
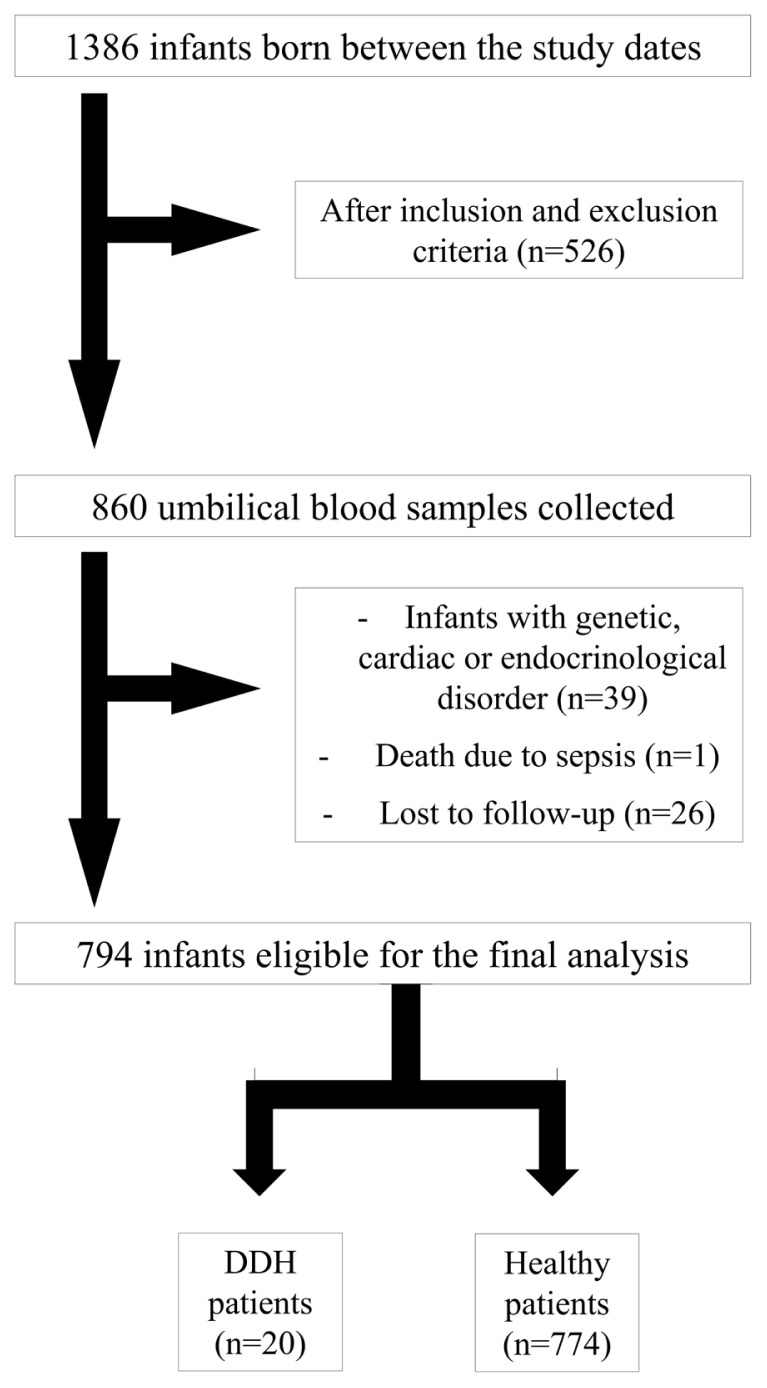
Diagram depicting the study population and the patients included in the final analysis.

**Table 1:** Descriptive and demographic characteristics of the study groups.

	Overall (n = 80)	Infants with DDH (n = 20)	Healthy infants (n = 60)	*p-value*

Birth weight (g)	3206.0 ± 344.9	3041.3 ± 481.6	3260.9 ± 269.0	0.065*
Mean ± SD				

Gestational age (days)	270.3 ± 7.3	267.9 ± 7.3	271.0 ± 7.2	0.098*
Mean ± SD				

Maternal age (years)	28.4 ± 5.6	29.7 ± 6.5	27.9 ± 5.3	0.222*
Mean ± SD				

Sex				
Male	58 (72.5%)	17 (85%)	41 (68.3%)	0.148+
Female	22 (27.5%)	3 (15%)	19 (31.7%)	

Delivery				
C/S	40 (50%)	9 (45%)	31 (51.7%)	0.606+
NVD	40 (50%)	11 (55%)	29 (48.3%)	

DDH type				
Type 1	60 (75.0%)	0 (0.0%)	60 (100.0%)	n/a
Type 2b	13 (16.3%)	13 (65.0%)	0 (0.0%)	
Type 2c	6 (7.5%)	6 (30.0%)	0 (0.0%)	
Type 3	1 (1.3%)	1 (5.0%)	0 (0.0%)	

Primigravida	63 (78.8%)	15 (75.0%)	48 (80.0%)	0.636+
Multiparity	17 (21.3%)	5 (25.0%)	12 (20.0%)	

+ Chi-squared,

* Mann–Whitney U test

C/S, cesarean section; NVD, normal vaginal delivery; DDH, developmental dysplasia of the hip; n/a, not applicable

**Table 2:** Comparison of cord blood IGF levels of healthy infants and those diagnosed with hip dysplasia.

Variable (ng/mL)	Infants with DDH Mean ± SD (n = 20)	Healthy infants Mean ± SD (n = 60)	*p-value* *
IGF-1	409.7 ± 115.3	440.9 ± 220.2	0.110
IGF-BP3	112.2 ± 42.1	132.9 ± 52.9	0.116
IGF-BP5	124.6 ± 16.9	143.6 ± 36.3	**0.028**

* Mann–Whitney U test

DDH, developmental dysplasia of the hip; IGF, insulin-like growth factor; IFG-BP, insulin-like growth factor-binding protein.

**Table 3:** Comparisons of cord blood IGF values and categorical study variables in infants with DDH.

	Infants with DDH		

	IGF-1	IGF-BP3	IGF-BP5

Sex			
Female	428.2 ± 112.8	113.9 ± 45.4	126.2 ± 17.0
Male	301.3 ± 60.8	102.3 ± 12.7	115.6 ± 16.7

*p-value*+	*0.093*	*0.146*	*0.358*

Primigravida	414.1 ± 124.7	105.8 ± 46.5	121.5 ± 18.6
Multiparity	396.6 ± 91.9	131.4 ± 15.7	134.2 ± 2.1

*p-value*+	*0.965*	*0.142*	*0.151*

DDH type			
Type 2b	454.6 ± 118.7	125.9 ± 24.0	119.8 ± 16.1
Type 2c	334.7 ± 33.9	100.3 ± 48.9	129.0 ± 11.3
Type 3	277.0 ± 0.0	5.0 ± 0.0	161.0 ± 0.0

p-value*	0.058	0.209	0.185

Side			
Bilateral	389.2 ± 135.4	107.3 ± 53.5	127.0 ± 18.0
Unilateral	427.1 ± 81.9	119.6 ± 14.2	121.1 ± 15.8

*p-value+*	0.208	0.970	0.464

* Kruskal–Wallis test,

+ Mann–Whitney U test

DDH, developmental dysplasia of the hip
